# Modeling hematopoietic disorders in zebrafish

**DOI:** 10.1242/dmm.040360

**Published:** 2019-09-01

**Authors:** Martina Konantz, Christoph Schürch, Pauline Hanns, Joëlle S. Müller, Loïc Sauteur, Claudia Lengerke

**Affiliations:** 1 Department of Biomedicine, University of Basel and University Hospital Basel, Basel 4031, Switzerland; 2 Division of Hematology, University of Basel and University Hospital Basel, Basel 4031, Switzerland

**Keywords:** Disease models, Hematopoiesis, Blood disorders, Leukemia, Immunodeficiency, Bone marrow failure syndrome

## Abstract

Zebrafish offer a powerful vertebrate model for studies of development and disease. The major advantages of this model include the possibilities of conducting reverse and forward genetic screens and of observing cellular processes by *in vivo* imaging of single cells. Moreover, pathways regulating blood development are highly conserved between zebrafish and mammals, and several discoveries made in fish were later translated to murine and human models. This review and accompanying poster provide an overview of zebrafish hematopoiesis and discuss the existing zebrafish models of blood disorders, such as myeloid and lymphoid malignancies, bone marrow failure syndromes and immunodeficiencies, with a focus on how these models were generated and how they can be applied for translational research.

## Abstract (precis)

**Summary:** This At A Glance article and poster summarize the last 20 years of research in zebrafish models for hematopoietic disorders, highlighting how these models were created and are being applied for translational research.

## Introduction

Zebrafish (*Danio rerio*) are increasingly used to study mechanisms regulating vertebrate tissue development and disease pathogenesis. Since especially blood cell types and their regulation are highly conserved (Box 1), many mutated zebrafish orthologs of human blood-disease-related genes have been successfully phenocopied, and the number of disease models is increasing with the current genomic advances. Additionally, specific advantages of the zebrafish model include its external fertilization and rapid development as well as (embryonic) transparency, facilitating *in vivo* imaging and the performance of genetic and small-molecule screens (Box 2) (Bertrand and Traver, 2009; Davidson and Zon, 2004; Li et al., 2015; Palis and Yoder, 2001). Since the first publication of the zebrafish genome in 2002 and its modifications and expansions in 2013, the zebrafish reference genome sequence has enabled many new discoveries, for example, the positional cloning of genes from mutations affecting embryogenesis, behavior and cell physiology in both healthy tissues and during disease pathogenesis (Howe et al., 2013). This review and accompanying poster summarize the current available hematopoietic disease models (see also Table 1), describes how they were generated and highlights their benefits.

**Figure:**
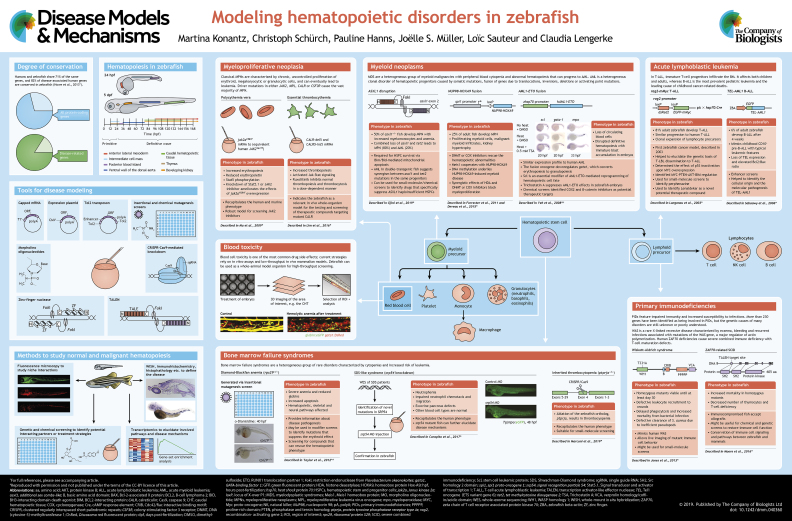

**Box 1. Hematopoietic development in zebrafish**

As in other vertebrates, zebrafish hematopoiesis develops in sequential waves (Davidson and Zon, 2004). Primitive hematopoiesis starts at two anatomically separate mesodermal sites in the embryo: the intermediate cell mass, which contributes to the first circulating erythrocytes, and the rostral blood island, which gives rise to primitive macrophages and neutrophils (Detrich et al., 1995; Palis and Yoder, 2001). A second transient hematopoietic wave occurs from the posterior blood islands, where multipotent erythromyeloid progenitors are generated between 24 and 30 hpf (Bertrand et al., 2007, 2010b). Between 28 and 32 hpf, definitive hematopoietic stem/progenitor cells (HSPCs) start emerging from the ventral dorsal aorta – the equivalent of the mammalian aorta-gonad-mesonephros region (Bertrand et al., 2010a, 2010b; Kissa and Herbomel, 2010). These definitive HSPCs then migrate to and amplify in the caudal hematopoietic tissue (Bertrand et al., 2010a; Boisset et al., 2010) – a site equivalent to the fetal liver in mammals – before they subsequently colonize the thymus and the kidney marrow. The latter is the adult hematopoietic organ and sustains hematopoiesis throughout the zebrafish life span (Chen and Zon, 2009; Jin et al., 2007), and the thymus enables T-cell maturation.

**Box 2. Methods, advantages and disadvantages for modeling hematological disorders in zebrafish**

Methods

**Transient strategies: mRNA or cDNA injections for overexpression of target genes, morpholino oligonucleotide (MO) injection for downregulation.** MOs are nonionic DNA analogs in which the ribose moiety has been substituted with an MO ring. They are generally designed to be complementary to the translational start site or a specific splice site in the pre-mRNA of the target gene, preventing translation or splicing of the pre-mRNA by a steric blocking mechanism. The technique is based on injecting these modified oligonucleotides, which then prevent expression of the targeted gene (see also https://www.gene-tools.com). Recently, serious concerns have been raised as to the specificity of MO effects (Kok et al., 2015). However, adequately controlled MOs used according to specific guidelines should still be accepted as a generic loss-of-function approach in the absence of genetic evidence (Blum et al., 2015; Stainier et al., 2017).

**Permanent strategies: transgene expression, which allows expression of human sequences or fusion reporters, and genome editing tools.** Zinc-finger nucleases (ZFNs) can be used for targeting a unique genomic locus. Transcription activator-like effector nucleases (TALENs) are suitable for knock-in strategies or for removing large spans of DNA to cause genomic deletions. TILLING (targeted induced local lesions in genomes) allows directed introduction of point mutations in a specific gene. The CRISPR (clustered regularly interspaced short palindromic repeats)/Cas9 system can cut at a specific location but also allows knock-ins or removal of existing genes/sequences (Phillips and Westerfield, 2014).

**Xenotransplantation of human cancer cells** (Konantz et al., 2012; Parada-Kusz et al., 2018; Veinotte et al., 2014) to generate patient-derived xenograft models, which may allow targeted therapy development and disease outcome prediction (Bentley et al., 2015; Gacha-Garay et al., 2019).

Advantages

High fecundity, small size and fast embryonic development, which make the zebrafish amenable for large-scale screens.

*Ex utero* fertilization and development, which allows (genetic) manipulation at all developmental stages and analyses of phenotypes that would die *in utero* in mice.

Transparency during development and in adult *casper* (White et al., 2008) or *tra/nac* (Krauss et al., 2013; Lister et al., 1999) fish, which allows live imaging of hematopoietic cells.

Conserved regulatory pathways, especially in hematopoiesis.

Disadvantages

Duplicated genome with many single-nucleotide polymorphisms and insertion/deletion variations.

Cold-blooded animal – evolutionarily far away from humans.

Lack of certain organs (e.g. lung, breast).

Lack of specific antibodies for experimental work.

Fish are greatly influenced by their environment (temperature, density etc.).

Different morphology of certain blood cells.

Lack of a fully functioning adaptive immune system.

**Table 1.**
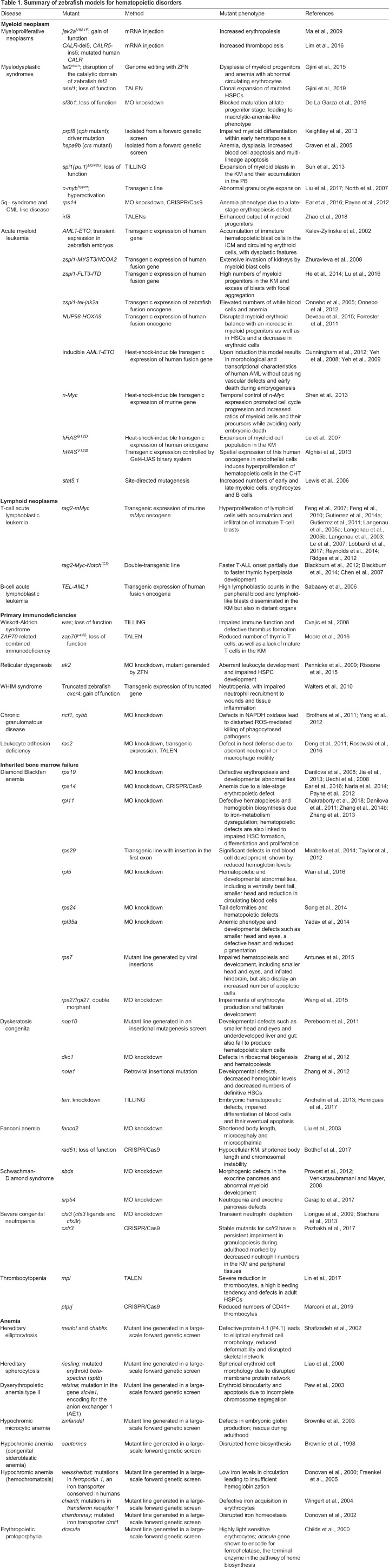
**Summary of zebrafish models for hematopoietic disorders**

Blood development is tightly regulated by complex interactions between hematopoietic stem cells (HSCs) and the microenvironment, making *in vivo* investigations mandatory. For human cells, these require analyses in xenograft models. These are naturally limited by incomplete interspecies protein cross-reactivity and the requirement for an immunosuppressed host animal to prevent graft rejection. Therefore, researchers have developed animal models for further *in vivo* assessment of genotype-phenotype relations in hematologic disorders. Here, we describe the currently available zebrafish models for hematopoietic disorders in more detail.

## Myeloid neoplasms

Myeloid malignancies are chronic or acute clonal diseases arising from hematopoietic stem and progenitor cells (HSPCs) characterized by uncontrolled proliferation and/or differentiation blocks in myeloid cells. Chronic myeloid neoplasms such as myeloproliferative neoplasms (MPNs), myelodysplastic syndromes (MDS) or chronic myelomonocytic leukemia (CMML) all have an increased risk of transformation into acute myeloid leukemia (AML) (Lindsley, 2017). The genetic causes for myeloid neoplasms are highly variable, but primarily occur in transcription factors, epigenetic regulators, tumor suppressors, signaling pathway proteins or components of the spliceosome. Many of these genes are essential for zebrafish blood development and have been successfully modeled to understand the underlying disease mechanisms.

### Myeloproliferative neoplasms

MPNs are classified into three subgroups – polycythemia vera (PV), essential thrombocythemia (ET) and primary myelofibrosis  – all of which are accompanied by disease-related complications, such as thrombosis and hemorrhages, and mainly affect people above 50 years of age (Vainchenker and Kralovics, 2017). Driver mutations in either *JAK2*, *MPL*, *CALR* or *CSF3R* (full names of genes/proteins used in this article are shown in Box 3) occur in the vast majority of MPN patients. Although treatment strategies exist, resistance to drugs such as JAK2 inhibitors remains a big challenge (Meyer, 2017).

**Box 3. Gene/protein symbols and names**

**ADP:** adenosine diphosphate

***AE1*:** anion exchanger 1

***AK2*:** adenylate kinase 2

***AKT*:** protein kinase B

***AML1*:** acute myeloid leukemia 1 gene; also known as *RUNX1*

**AMP:** adenosine monophosphate

***asxl1*:** *additional sex combs like*

**ATG5:** autophagy protein 5

**ATP:** adenosine triphosphate

***BCL2*:** B-cell lymphoma 2

***BIM*:** Bcl2-interacting protein

**CALR:** calreticulin

**Cas9:** caspase 9

***c-myb*:** *myb proto-oncogene*

**COX:** cyclooxygenase

**Cre:** cAMP response element

***CSF3R*:** colony-stimulating factor 3 receptor, granulocyte

***CXCR4*:** CXC chemokine receptor 4

**DKC1:** dyskerin

**DMT1:** FTD3 frontotemporal dementia, chromosome 3-linked

**ER:** estrogen receptor

***ETO*:** RUNX1 translocation partner 1

***ETV5*:** ETS variant gene 5

***EZH2*:** enhancer of zeste, Drosophila, homolog 2

***fancd2*:** *Fanconi anemia, complementation group D2*

***FLT3*:** Fms-related tyrosine kinase 3

**Gal4:** Gal4 transcription factor

***gar1*:** *H/ACA ribonucleoprotein complex subunit 1*

***GCSF*(*R*):** granulocyte colony stimulating factor (receptor)

**GFP:** green fluorescent protein

**HOX:** homeobox transcription factor

**HRAS:** V-HA-RAS Harvey rat sarcoma viral oncogene homolog

***hspa9b*:** *heat-shock 70-kD protein 9 variant b*

***irf8*:** *interferon regulatory factor 8*

***JAK2*:** Janus kinase 2

**KRAS:** V-KI-RAS2 Kirsten rat sarcoma viral oncogene homolog

***lck*:** *lymphocyte-specific protein-tyrosine kinase*

**LEF1:** lymphoid enhancer-binding factor 1

***lmo2*:** *LIM domain only protein 2*; encodes Rhombotin-like 1

***MPL*:** myeloproliferative leukemia virus oncogene

***MYST3*:** histone acetyltransferase KAT6A

**mTOR:** mechanistic target of rapamycin

**NADPH:** nicotinamide adenine dinucleotide phosphate

***NCOA2*:** nuclear receptor co-activator 2

***n-Myc*:** v-Myc avian myelocytomatosis viral-related oncogene, neuroblastoma-derived

***nola1*:** *nucleolar protein family A, member 1*

***nop10:*** *H/ACA ribonucleoprotein complex subunit 3*

***NUP98*:** nucleoporin 98

***prpf8*:** *precursor mRNA-processing factor*

**PTEN:** phosphatase and tensin homolog

**PTPRJ:** receptor-type tyrosine-protein phosphatase eta

***RAC2*:** Ras-related C3 botulinum toxin substrate 2

***rad51*:** *DNA repair protein RAD51 homolog 2*

***rag2*:** *recombination-activating gene 2*

**RPL:** ribosomal protein L

**RPS:** ribosomal protein

***RUNX1*:** Runt-related transcription factor 1

***scl*:** *stem cell leukemic protein*

***sf3b1*:** *splicing factor 3B, subunit 1*

***slc4a1*:** *solute carrier family 4 (anion exchanger), member 1*

***spi1*:** *spleen focus forming virus proviral integration oncogene*

***sptb*:** *erythroid beta-spectrin*

***SRP54*:** signal recognition particle 54

***stat5.1*:** *signal transducer and activator of transcription 1*

***syk*:** *spleen tyrosine kinase*

**TCR:** T-cell receptor

***TEL*:** TEL1 oncogene

***TERT*:** telomerase reverse transcriptase

***TET2*:** Tet methylcytosine dioxygenase 2

**TOX:** thymocyte selection-associated high mobility group box protein

***tp53*:** *cellular tumor antigen* *p53*

***ZAP70*:** zeta chain of T cell receptor associated protein kinase 70

A major defining genetic event in human MPN is a gain-of-function mutation (V617F) in the *JAK2* gene (Baxter et al., 2005; James et al., 2005; Kralovics et al., 2005; Vainchenker and Kralovics, 2017). To model this disease in zebrafish, an ortholog of human *JAK2^V617F^* was created by site-directed mutagenesis (see poster: Myeloproliferative neoplasia). The mutant had a high degree of similarity to human PV, mainly characterized by erythroid expansion (Ma et al., 2009). Another gene commonly mutated in MPN patients without *JAK2^V617F^* is *CALR*, which encodes the endoplasmic reticulum chaperone calreticulin. Expression of mutated human *CALR* in zebrafish embryos by mRNA injection caused an increase in thrombopoiesis via Jak/Stat signaling upregulation, resembling the phenotype observed in ET patients (see poster: Myeloproliferative neoplasia) (Lim et al., 2016). Both lines provide robust models for screening for therapeutic agents targeting Jak/Stat signaling. An accurate zebrafish model for primary myelofibrosis has not yet been developed.

### Myelodysplastic syndromes

Owing to their heterogeneity, MDS are particularly challenging to accurately model in animals. Mutations in genes associated with myeloid malignancies or pre-malignancy [clonal hematopoiesis of indeterminate potential (CHIP)] (Heuser et al., 2016) and especially mutations of epigenetic or splicing factors are commonly detected in MDS, either alone or in various combinations. One of the genes most commonly associated with CHIP and myeloid malignancies is *TET2*, an epigenetic factor regulating DNA methylation. Somatic loss-of-function *tet2^m/m^* zebrafish mutants engineered by zinc-finger nuclease (ZFN) genome editing develop normally during embryogenesis, but show progression to clonal myelodysplasia as they age and eventually develop MDS-like features at 24 months post-fertilization (Gjini et al., 2015). Subsequently, the same group generated an *asxl1* mutant (see poster: Myeloid neoplasms). Somatic loss-of-function mutations of this gene are common genetic abnormalities in human myeloid malignancies and induce clonal expansion of mutated HSPCs. The authors showed that half of the heterozygous fish developed MPN by 5 months of age. Interestingly, the combination of heterozygous loss of *asxl1* with heterozygous loss of their previously generated *tet2* mutant led to a more penetrant phenotype, while *asxl1^+/−^* together with complete loss of *tet2* even caused AML (Gjini et al., 2019).

In another recent model, a loss-of-function mutation of *sf3b1* in zebrafish leads to spliceosomal defects and thus MDS-like phenotypes (De La Garza et al., 2016). Furthermore, the *cephaloponus* mutant, which was isolated from a forward genetic screen followed by a positional cloning scan, showed that its driver mutation was affecting the splicing factor gene *prpf8* (Keightley et al., 2013). Another mutant identified in a forward genetic screen is *crimsonless*, which represents one of the very first zebrafish MDS models and was shown to carry a mutation in a gene encoding a ubiquitously expressed matrix chaperone, *hspa9b* (Craven et al., 2005). Next to these approaches, targeting induced local lesions in genomes (TILLING) is a reverse genetic method that enabled the association of *spi1* loss of function with MDS development (Sun et al., 2013). Furthermore, a rather unusual but promising zebrafish model for MDS is the *c-myb^hyper^* strain, initially developed as a *Tg(c-myb:GFP)* reporter line (North et al., 2007). Liu and colleagues, however, discovered that the transgene causes hyperactivation of *c-myb* by expressing an alternative transcript lacking the negative regulatory domain; this *c-myb* hyperactivation eventually led to MDS that progresses to transplantable AML and acute lymphoblastic leukemia (ALL) (Liu et al., 2017), and thus provides a promising model for future drug screenings.

### 5q– syndrome and CML-like disease

5q– syndrome is a distinct form of MDS caused by a deletion on chromosome 5. Patients with this syndrome suffer from macrocytic anemia with other hematological phenotypes (i.e. thrombocytosis and megakaryocyte hyperplasia). Ear and colleagues used clustered regularly interspaced short palindromic repeats (CRISPR)/Cas9 to target *rps14* by introducing an early stop codon via non-homologous end joining (Ear et al., 2016). This technology has revolutionized genome editing and massively facilitated the engineering of animal models of disease (Boel et al., 2018; Prykhozhij et al., 2017). Targeted mutation of *rps14* indeed led to anemic defects resembling those seen in 5q– syndrome (Ear et al., 2016), which was already modeled by morpholino oligonucleotide (MO) knockdown in a previous study (Payne et al., 2012). Besides, researchers also used transcription activator-like effector nucleases (TALENs) to generate mutations in the *irf8* gene in zebrafish, which – as in mice – causes a type of MPN known as chronic myeloid leukemia (CML)-like disease (Holtschke et al., 1996; Zhao et al., 2018).

### Acute myeloid leukemia

AML is defined as acute malignant disease characterized by uncontrolled proliferation and accumulation of leukemic blasts in the bone marrow (BM), peripheral blood (PB) and other organs (Ferrara and Schiffer, 2013; Greim et al., 2014). It is the most common type of acute leukemia in adults and can occur at all ages, but more frequently affects elderly people, where it mainly progresses with an aggressive clinical course (Herrmann et al., 2012; Juliusson et al., 2009; Mrózek et al., 2012). Although the outlook for AML patients has improved over recent decades, more than half of young-adult and about 90% of elderly patients die from the disease (Mrózek et al., 2012). The main obstacles to cure are refractoriness to initial induction treatment and, more frequently, relapse after apparent remission.

One of the first AML models in zebrafish involved the transient expression of the human fusion oncogene *AML1* (*RUNX1*)*-ETO* in zebrafish embryos. This disrupted normal hematopoiesis, with accumulation of immature hematopoietic blast cells in the intermediate cell mass (ICM), and circulating erythroid cells with dysplastic features (Kalev-Zylinska et al., 2002). Following this model, several others were developed, predominantly through the (over)expression of fusion oncogenes (Tan et al., 2018), and furthermore demonstrated potential for drug screenings. However, although these models enabled extensive studies on embryonic phenotypes, they associated with early embryonic lethality and were thus not suitable for studies in adult animals. The first successful zebrafish model of stable and embryonic non-lethal AML was established by Zhuravleva et al. (2008). It featured transient expression of a fusion of the human histone acetyl-transferase *MYST3* with *NCOA2* under the control of the myeloid-specific *spi1* promoter. A small number of transgenic embryos expressing the fusion transgene presented 14-26 months later with myeloid blast expansion in the kidney marrow (KM), as is commonly observed in human AML. Owing to its specificity to early myeloid lineages, *spi1*-driven oncogene expression was used in several additional myeloid malignancy models, e.g. involving the oncogenic fusion proteins *FLT3*-ITD [internal tandem duplication (ITD) of FLT3; He et al., 2014; Lu et al., 2016), *tel-jak2a* (CML) (Onnebo et al., 2005, 2012) and *NUP98-HOXA9* (see poster: Myeloid neoplasms) (Deveau et al., 2015; Forrester et al., 2011). Interestingly, the latter led to the identification of specific epigenetic therapies that restore healthy hematopoiesis in *NUP98-HOXA9* fish and of synergistic effects between DNA methyltransferase and cyclooxygenase inhibitors (Deveau et al., 2015).

An alternative way to overcome embryonic lethality upon human oncogene expression in zebrafish is to make use of temporal and spatial promoter activity by heat-shock treatment combined with Cre-mediated induction. Yeh et al. developed a heat-shock-inducible *AML1-ETO* model (see poster; Myeloid neoplasms), which, upon induction, resulted in morphological and transcriptional characteristics of human AML without causing vascular defects and early death during embryogenesis (Yeh et al., 2008). Interestingly, expression profiles of these fish resemble those seen in human AML, and the authors found *scl* to be an essential modifier of the ability of *AML1-ETO* to reprogram hematopoietic cell fate decisions (Yeh et al., 2008). A subsequent modifier screen surprisingly exposed roles of COX2- and β-catenin-dependent pathways in *AML1-ETO* function (Yeh et al., 2009). In another heat-shock-inducible system, Shen et al. timed expression of the murine *n-Myc* and thereby succeeded in inducing myeloid defects while avoiding early embryonic death. Specifically, *n-Myc* promoted cell cycle progression and increased the ratios of myeloid cells and their precursors (Shen et al., 2013). Following the same principle of timed heat-shock induction, Le and colleagues showed KRAS^G12D^-associated myeloid cell expansion in the KM (Le et al., 2007). A different approach of selective oncogene expression was exemplified by Alghisi and colleagues, who used the Gal4-UAS (upstream activated sequence) binary system (Scheer and Campos-Ortega, 1999) to express HRAS^V12G^ specifically in endothelial cells, which induced hyperproliferation of hematopoietic cells in the caudal hematopoietic tissue (CHT) (Alghisi et al., 2013). Remarkably, the authors showed that the abnormal phenotype in their model was associated with downregulation of the Notch pathway, which could be rescued by Notch overexpression in endothelial cells. Other models involve constitutive activation of *stat5.1* (Lewis et al., 2006) or expression of known mutations involved in myeloid neoplasms (Barbieri et al., 2016; Bolli et al., 2010; Shi et al., 2015; Zhao et al., 2018). Although these models provide opportunities for further research, most of them do not fully recapitulate the features of human AML. In fact, some of these models might represent pre-leukemic stages, probably because they are based on a single genetic manipulation, while human leukemogenesis requires several genetic alterations. Owing to recent technological advances in genome editing, and especially to the generation of efficient inducible promoters that circumvent early embryonic lethality, it may soon be possible to simultaneously manipulate multiple genes within the same cell lineage and to thereby obtain more robust leukemia models.

## Lymphoid neoplasms

ALL is a malignant disorder of lymphoid progenitor cells affecting both children and adults. It can be separated into T-cell acute lymphoblastic leukemia (T-ALL) and B-cell acute lymphoblastic leukemia (B-ALL). Multi-agent combination chemotherapy regimens exist and result in cure rates of >90% for children and 40% for adults (Dinner and Liedtke, 2018).

### T-cell acute lymphoblastic leukemia

T-ALL is characterized by immature T-cell-progenitor infiltration in the BM and accounts for 15% of ALL cases in pediatric patients and 25% of ALL in adults (Dinner and Liedtke, 2018). Mutations and rearrangements in several genes have been implicated in T-ALL, such as in HOX genes, genes regulating RAS signaling (e.g. *FLT3*), histone-modifying genes (e.g. *EZH2*), transcription-factor tumor suppressors (e.g. *AML1*, *ETV5* or *LEF1*), mutations affecting the *NOTCH1* pathway, and many more. In many T-ALL cases, either *MYC* or *MYC-n* are upregulated, suggesting the *MYC* pathway as a central regulator of T-ALL in humans. The majority of the reported ALL zebrafish models show a T-ALL phenotype, and transgenic *rag2-mMyc* zebrafish were the first cancer models described in zebrafish (Langenau et al., 2003). This is mainly due to the use of the lymphoid cell promoter *rag2* to drive specific oncogenic expression. Although involved in both T-ALL and B-ALL development in zebrafish (Borga et al., 2019; Garcia et al., 2018), all early *rag2*-driven ALL models developed in the 2000s exclusively induced T-cell neoplasia. Leukemias convincingly presented with hyperproliferation of lymphoid cells with accumulation and infiltration of immature T-cell blasts in various tissues and organs. The commonly used oncogene in these models is *c-Myc*. Various different *rag2:Myc* models have been described, mainly differing in the way the oncogene is expressed. The initial T-ALL model described in 2003 was exclusively propagated by *in vitro* fertilization due to premature lethality (Langenau et al., 2003). Later on, the use of inducible promoters overcame early lethality. Langenau et al. used a Cre-inducible model (Langenau et al., 2005a) and Gutierrez et al. established conditional tamoxifen-inducible *rag2:Myc-ER* fish, which allowed improved analyses and assessed direct causality between *Myc* oncogene expression and T-ALL (Gutierrez et al., 2011). Interestingly, all Myc-induced T-ALL models follow a similar disease progression pattern, starting with localized T-lymphoblastic lymphoma with minor outgrowth before disseminating into the circulation and infiltrating other tissues with T-ALL-like cells (see poster; Acute lymphoblastic leukemia) (Feng et al., 2007; Langenau et al., 2005b, 2008; Rudner et al., 2011). The similarities between zebrafish and mammalian Myc-induced T-ALL enabled detailed analyses of the mechanisms underlying leukemic transformation (Blackburn et al., 2014; Feng et al., 2010; Reynolds et al., 2014). As such, and in line with the expression patterns observed in subtypes of human T-ALL (Langenau et al., 2005a), the effect of p53 inactivation during Myc-induced T-ALL onset could be determined by zebrafish studies (Feng et al., 2007, 2010; Gutierrez et al., 2014a). Additionally, researchers dissected the MYC-PTEN-AKT-BIM pathway in zebrafish, which demonstrated that PTEN-inactivating mutations promote loss of *MYC* oncogene dependence, and upregulation of the oncogenes *scl* and *lmo2* was found in Myc-induced cells in zebrafish (Gutierrez et al., 2011, 2014a; Reynolds et al., 2014). Notably, these lines were used to identify novel players and compounds for T-ALL treatment. In an attempt to identify compounds with selective toxicity against ALL, Ridges and colleagues used transgenic *Tg(lck:eGFP)* fish for a small-molecule screen and then confirmed hits in tamoxifen-inducible *rag2:Myc-ER* animals. They identified Lenaldekar, which is an active compound against immature normal and MYC-transformed leukemic T cells in adult zebrafish (Ridges et al., 2012). In another screen, phenothiazines were identified as compounds with NOTCH-independent anti-T-ALL activity (Gutierrez et al., 2014b). Additionally, researchers found TOX in a transgenic screen, which regulates growth, DNA repair, and genomic instability in T-ALL (Lobbardi et al., 2017).

Another central oncogene associated with T-ALL is *NOTCH1*. *rag2*-driven expression of the Notch1 intracellular domain (ICN1) causes constitutive activation of Notch signaling in T cells, eventually leading to the development of T-ALL in zebrafish (Blackburn et al., 2012; Chen et al., 2007). The combination of constitutive Notch activation with expression of the anti-apoptotic molecule *bcl2* further increased T-ALL incidence and accelerated manifestation with an earlier disease onset than with Notch activation alone (Chen et al., 2007). Later studies showed that Notch, which was thought to mainly exert its oncogenic function through transcriptional activation of *Myc*, also acts via *Myc*-independent mechanisms. However, Notch activation alone only leads to the expansion of a pre-malignant thymocyte pool without affecting the overall number of leukemia propagating cells (Blackburn et al., 2014).

### B-cell acute lymphoblastic leukemia

B-ALL is a hematologic malignancy derived from immature B-cell precursors. It is the most prevalent childhood leukemia and the leading cause of childhood cancer-related deaths. B-ALL can be divided into several subtypes, including pro-B, pre-B, common and mature B-ALL. Although 75% of human ALL cases are B-ALL, modelling this disease in zebrafish is difficult due to the T-cell bias of the *rag2* promoter. Until recently, only one model of pre-B-ALL induction through global expression of the fusion oncogene *TEL-AML1* has been described (see poster; Acute lymphoblastic leukemia) (Sabaawy et al., 2006). However, the low incidence and the long latency of leukemia development in this model suggests that acquisition of additional mutations is most likely necessary to induce leukemic transformation. A recent promising and surprising discovery was the development of coincident B-ALL in *rag2*-driven *Myc* models, which were before considered to be T-ALL specific. Borga et al. used a tissue-specific reporter line (*Tg(lck:eGFP)*), which differentially labels B and T cells, and observed clustering of *rag2*-induced *hMYC* ALL models according to the overall GFP intensity. Intensive investigation of the different clusters revealed the expression of B-cell-specific genes – predominantly in low-GFP-expressing ALL cells – and the development of pre-B-ALL (Borga et al., 2019). At the same time, another group discovered B-ALL features in a subset of *Tg(rag2:mMyc)* zebrafish by propagating ALL via single-cell allotransplantation followed by single-cell transcript expression (Garcia et al., 2018). These novel findings may represent an alternative way of using the *rag2* promoter to establish B-ALL zebrafish models.

## Primary immunodeficiencies

Primary immunodeficiencies (PIDs) comprise all disorders that feature impaired immunity, which often leads to increased susceptibility to infections (Raje and Dinakar, 2015). The most dangerous forms of PID are severe combined immunodeficiencies (SCID). This subgroup is characterized by a block in T-cell differentiation associated with an additional defect in any other immune cell lineage (Fischer, 2000).

### Wiskott-Aldrich syndrome

Wiskott-Aldrich syndrome (WAS) is caused by mutations in the X-linked *WAS* gene, which encodes the WAS protein (WASp). WASp is only produced in hematopoietic cells and plays a central role in transmitting cell-surface signals to the actin cytoskeleton. Several different inactivating mutations of *WAS* manifest in eczema, microthrombocytopenia and recurrent infections, and the severity of symptoms correlates with the degree of WASp loss (Massaad et al., 2013). Cvejic et al. performed detailed live-imaging experiments on zebrafish *was* morphants and loss-of-function mutants that they generated by TILLING. They observed impaired innate immune function associated with defective thrombus formation (Cvejic et al., 2008). Later, the same lab used the Gal4/UAS system to dissect the function of different human *WAS* mutant alleles by targeting their expression specifically to neutrophils and macrophages in WASp-null zebrafish (see poster: Primary immunodeficiencies) (Jones et al., 2013).

### *ZAP70*-related combined immunodeficiency

*ZAP70*-related combined immunodeficiency (CID) is the rarest form of SCID, with around 50 known affected individuals. A mutation in *ZAP70* leads to abnormal TCR signaling, resulting in the absence of peripheral CD8^+^ and non-functional CD4^+^ T cells. Furthermore, the absence of T cells facilitates impaired immunoglobulin production in B cells (Arpaia et al., 1994; Elder, 1996; Elder et al., 1994, 1995). Zebrafish models have been extensively used to study ZAP70 deficiency and a possible compensatory mechanism by *syk*. Whilst research on the first knockdown models mainly focused on vascular development (Christie et al., 2010), a mutant developed by TALENs successfully recapitulated the immune defects seen in humans (see poster; Primary immunodeficiencies) (Moore et al., 2016).

### Reticular dysgenesis

Patients suffering from reticular dysgenesis (RD) commonly present with SCID in combination with agranulocytosis and sensorineural deafness. The underlying genetic cause of RD is mutations in the *AK2* gene, encoding for adenylate kinase 2, which catalyzes the phosphotransfer from ATP to AMP, resulting in ADP production (Dzeja et al., 1998). Currently, HSC transplantation is the only option to treat RD patients (Hoenig et al., 2017). Morpholino knockdown was performed to mimic RD in zebrafish (Pannicke et al., 2009) and data from this study were recently confirmed by Rissone and colleagues, who aimed to generate a variety of different *ak2* mutations, as seen in humans, and thus analyzed a loss-of-function *ak2* mutant from a DNA library of N-ethyl-N-nitrosourea (ENU)-induced mutations (Sood et al., 2006) and furthermore generated a knockout (KO) model for *ak2* by using ZFNs to introduce targeted frameshift mutations in the first exon (Rissone et al., 2015).

### WHIM syndrome

Myelokathexis is a rare disorder with recurrent bacterial infections caused by a reduced number and function of neutrophils. WHIM syndrome refers to the association of features from which its name derives, including warts, hypogammaglobulinemia and infections with myelokathexis. In most patients, WHIM arises from gain-of-function mutations in *CXCR4* (Kawai and Malech, 2009). To model the disease in zebrafish, a truncated version of *CXCR4* was stably expressed in neutrophils. Whole-mount *in situ* hybridization and live imaging of these fish revealed a high degree of similarity to WHIM phenotypes observed in patients (Walters et al., 2010).

### Chronic granulomatous disease

CGD is an inherited PID characterized by dysregulated inflammation, autoimmunity and severe infections caused by defects of the NADPH oxidase complex in neutrophilic granulocytes and monocytes (Arnold and Heimall, 2017). In zebrafish, different morphants demonstrated the necessity of a functional NADPH oxidase complex for reactive oxygen species (ROS)-mediated killing of phagocytosed pathogens (Brothers et al., 2011; Harvie and Huttenlocher, 2015; Yang et al., 2012). However, no stable zebrafish model for CGD has been established yet.

### Leukocyte adhesion deficiency

Leukocyte adhesion deficiency (LAD) syndromes are rare PIDs characterized by adhesion-dependent malfunctions of leukocytes. Until now, three different subtypes of LAD have been described (LAD I-III). LAD-I is characterized by absent or reduced expression of β2 integrins, LAD-II is hallmarked by defects in fucosylation of selectin ligands and LAD-III patients suffer from defects in integrin signaling (Harris et al., 2013). Owing to the aberrant adhesion properties, all LAD patients have increased numbers of circulating neutrophils. Huttenlocher and co-workers established a zebrafish model mimicking phenotypes observed in LAD patients by mutating *rac2*, a Rho GTPase largely restricted to hematopoietic cells. *rac2* morphants, zebrafish expressing mutated *rac2* in neutrophils, or *rac2* TALEN knockouts all present with defects in host defense due to aberrant neutrophil or macrophage motility (Deng et al., 2011; Rosowski et al., 2016). However, several phenotypes observed upon human *RAC2* deficiency, such as altered polarity and mobilization from the CHT, were missing in the zebrafish KO models, indicating that alternative *rac2* isoforms may contribute to the phenotypic manifestation.

## Inherited bone marrow failure syndromes

Inherited BM failure syndromes (IBMFS) are a heterogeneous group of rare disorders characterized by BM failure resulting in cytopenias and increased risk of leukemia development (Dokal and Vulliamy, 2010). Many IBMFS have been successfully reconstituted in zebrafish (Oyarbide et al., 2019).

### Diamond-Blackfan anemia

Diamond-Blackfan anemia (DBA) is a genetically very heterogeneous sporadic disorder. Although its main characteristic is erythrocyte aplasia that normally presents before 1 year of age, it is accompanied by a wide variety of phenotypic anomalies, such as skeletal deformations and short stature (Diamond et al., 1961; Engidaye et al., 2019; Ito et al., 2010). More than 50% of DBA patients carry mutations in genes encoding ribosomal proteins (Taylor and Zon, 2011; Vlachos and Muir, 2010). The first zebrafish models of DBA were established in 2008 by two different laboratories, both using MO injection to knock down *rps19*. The knockdown led to DBA-like phenotypes hallmarked by defective erythropoiesis and developmental abnormalities (Danilova et al., 2008; Jia et al., 2013; Uechi et al., 2008). These findings rapidly triggered the establishment of numerous novel ribosomal-protein-driven DBA models, such as *rps14* (Narla et al., 2014), *rpl11* (Chakraborty et al., 2018; Danilova et al., 2011; Zhang et al., 2013, 2014b), *rps29* (Mirabello et al., 2014; Taylor et al., 2012) (see poster: Bone marrow failure syndromes), *rpl5* (Wan et al., 2016), *rps24* (Song et al., 2014), *rpl35a* (Yadav et al., 2014), *rps7* (Antunes et al., 2015), *rps27/rpl27* (Wang et al., 2015) and *rps11* (Zhang et al., 2014a). Most of these were first developed using MO knockdown and later established as stable transgenic zebrafish lines, predominantly by using TALENs. A common finding in all models was the upregulation of the p53 pathway upon ribosomal protein deficiency. However, simultaneous knockdown of *tp53* was not able to completely rescue BM defects, indicating the involvement of p53-independent mechanisms (Antunes et al., 2015; Chakraborty et al., 2018; Danilova et al., 2008; 2011; Torihara et al., 2011; Wan et al., 2016; Yadav et al., 2014; Zhang et al., 2013, 2014a). Interestingly, treatment of DBA embryos with an exogenous supply of nucleosides resulted in downregulation of *tp53*, reduced apoptosis and rescue of hematopoiesis (Danilova et al., 2014). Furthermore, it has recently been suggested that the immune system might be involved in the pathophysiology of DBA. Using two models (*rpl11* mutants and *rps19* morphants), Danilova and colleagues showed upregulation of interferons, inflammatory pathways and the complement system in DBA zebrafish models (Danilova et al., 2018). Remarkably, Payne and others could show that the amino acids L-leucine (Narla et al., 2014; Payne et al., 2012; Yadav et al., 2014) and L-arginine improve DBA symptoms via the mTOR pathway. This has led to a first clinical pilot phase I/II study of leucine in the treatment of DBA patients (https://clinicaltrials.gov/ct2/show/NCT01362595). Moreover, SMER28 (6-bromo-N-2-propenyl-4-quinazolinamine), a small-molecule inducer of ATG5-dependent autophagy, has been identified in a screen using DBA induced pluripotent stem cells and was confirmed in zebrafish models (Doulatov et al., 2017), highlighting the fact that zebrafish are a valuable model for drug identification and screening.

### Dyskeratosis congenita

Dyskeratosis congenita (DC) is a rare inherited disorder phenotypically characterized by BM failure, mucocutaneous abnormalities and premature aging. Genetically, DC patients almost exclusively present with mutations linked to the H/ACA ribonucleoprotein complex or telomere maintenance, thus often carrying shortened telomeres (Nelson and Bertuch, 2012). In 2011, Pereboom and colleagues described a zebrafish mutant that developed a DC-like phenotype (Pereboom et al., 2011). The mutant was generated in a large-scale insertional mutagenesis screen and featured viral insertion in the *nop10* gene, resulting in decreased transcript levels (Amsterdam et al., 1999). Nop10 is a dual-function protein involved in 18S ribosomal RNA (rRNA) processing and in the telomerase complex. Its knockdown in zebrafish resulted in ribosome biogenesis defects eventually leading to cytopenia. The most common and most severe form of DC is the X-linked form caused by mutations in *DKC1*, encoding the protein dyskerin. Dyskerin is a subunit of the H/ACA ribonucleoprotein complex and zebrafish *dkc1* mutants showed defects in ribosomal biogenesis and hematopoiesis. In the same study, a retrovirally mutated *nola1* zebrafish strain, which encodes for *gar1* and plays crucial roles in rRNA maturation and telomerase activity, developed similar phenotypes to *dkc1* mutants. Surprisingly, none of these models developed telomere defects (Zhang et al., 2012). Another gene commonly mutated in DC patients is *TERT*, which encodes the reverse transcriptase subunit of the telomerase complex. Three different studies described a zebrafish *tert^−/−^* mutant with disrupted tissue homeostasis and premature aging, thus representing a model for telomere shortening and disease anticipation in DC; however, it lacked classical symptoms such as BM failure and mucocutaneous abnormalities (Anchelin et al., 2013; Carneiro et al., 2016; Henriques et al., 2017).

### Fanconi anemia

Fanconi anemia (FA) is an autosomal recessive disorder manifesting with BM failure associated with other syndromic malformations such as skeletal defects and an increased risk of malignant transformation (Oyarbide et al., 2019; Tischkowitz and Hodgson, 2003). The genetic background of FA includes known mutations in different FA pathway genes, which are required for efficient DNA repair (Bagby, 2018). Two different zebrafish models for FA have been published so far. The first is a *fancd2* morphant whose phenotype resembles that observed in children suffering from FA, hallmarked by shortened body length, microcephaly, and microopthalmia due to an increase in spontaneous chromosomal breakage (Liu et al., 2003). The second model is a loss-of-function mutant of the DNA recombination gene *rad51*. Similar to the *fancd2* morphant, *rad51* loss of function leads to the development of an FA-like phenotype including hypocellular KM, shortened body length and chromosomal instability (Botthof et al., 2017).

### Shwachman-Diamond syndrome

Shwachman-Diamond syndrome (SDS) is a rare multisystem disorder that belongs to the severe congenital neutropenia (CN) group of disorders. It is characterized by exocrine pancreatic insufficiency, skeletal abnormalities and hematopoietic defects, with most patients suffering from neutropenia and increased risk of leukemic transformation. In total, 90% of SDS patients carry mutations in the Shwachman-Bodian-Diamond syndrome (*SBDS*) gene, which encodes a protein essential for ribosome biogenesis (Burroughs et al., 2009). The zebrafish *sbds* gene has been successfully knocked down by MO injection. Morphant fish developed a phenotype highly similar to that of SDS patients, with morphogenic defects in the exocrine pancreas and abnormal myeloid development (Provost et al., 2012; Venkatasubramani and Mayer, 2008). Recently, mutations in *SRP54* were described as being associated with SDS-like phenotypes or CN in patients (Bellanné-Chantelot et al., 2018; Carapito et al., 2017). An *srp54*-knockdown zebrafish model was established by Carapito and Konantz and colleagues that revealed that suppression of *srp54* induces neutropenia and exocrine pancreas defects in zebrafish embryos (see poster: Bone marrow failure syndromes) (Carapito et al., 2017).

### Severe congenital neutropenia

CN describes a heterogeneous group of hematological disorders that share the common feature of an absolute neutrophil count below 0.5×10^9^/L and increased incidence of infections in most patients. Around 60-80% of CN patients carry mutations in the neutrophil elastase gene (*ELA2/ELANE*) (Skokowa et al., 2017; Welte and Zeidler, 2009). *csf3* ligands and *csf3r* [zebrafish homologs of granulocyte colony stimulating factor and its receptor (*GCSF/R*)] are known to regulate and maintain neutrophil numbers during primitive and definitive hematopoiesis as shown by MO-mediated knockdown experiments (Liongue et al., 2009; Stachura et al., 2013). Various groups furthermore demonstrated that mutations in CSF3R lead to severe CN (e.g. Klimiankou et al., 2015; Triot et al., 2014). Pazhakh and colleagues therefore used CRISPR/Cas9 targeting to develop stable transgenic lines in zebrafish that maintained neutropenia in adulthood (Pazhakh et al., 2017), serving as a new animal model of human CSF3R-dependent CN.

### Thrombocytopenia

Like CNs, thrombocytopenias describe a variety of heterogeneous disorders. In humans, thrombocytopenia is defined by a platelet count of less than 150×10^3^/μl (Gauer and Braun, 2012). A zebrafish model for congenital amegakaryocytic thrombocytopenia was developed by mutating the *mpl* gene with TALENs (Lin et al., 2017). Recently, Marconi and colleagues identified loss-of-function variants of *PTPRJ* in inherited thrombocytopenia patients without a known genetic background. Ablation of zebrafish *ptprja* by CRISPR/Cas9 successfully recapitulated the patient phenotypes in zebrafish (see poster: Bone marrow failure syndromes) (Marconi et al., 2019).

## Anemia

Several forms of anemia (a reduction of erythrocytes) have been modeled in zebrafish. Genetic anemia models were mainly identified in large-scale genetic screens in the 1990s and later cloned and characterized (Driever et al., 1996; Haffter et al., 1996; Ransom et al., 1996). Hereditary elliptocytosis (HE) and hereditary spherocytosis (HS), two forms of hemolytic anemia that are caused by abnormal membrane cytoskeleton, for example, were reconstituted in zebrafish from mutants originally generated in one of these large-scale screens. The *merlot* and *chablis* strains share common features of HE, which, as shown by Shafizadeh et al., is due to protein 4.1 (P4.1) deficiency. As in HE patients, P4.1 defects led to elliptical erythroid cell morphology, reduced cell deformability and disrupted skeletal network (Shafizadeh et al., 2002). Another mutant called *riesling* was identified as a model for HS, as it carries a mutation in *sptb*, which as in humans results in spherical erythroid cell morphology due to disrupted membrane protein network (Liao et al., 2000). The zebrafish mutant *retsina* represents a model for dyserythopoietic anemia type II. The driver mutation in *retsina* is in the *slc4a1* gene encoding for the anion exchanger AE1, eventually resulting in erythroid binocularity and apoptosis due to incomplete chromosome segregation (Paw et al., 2003).

Furthermore, various zebrafish models for hypochromic anemia exist. Hypochromic anemia is characterized by pale and small erythrocytes, normally caused by globin or iron deficiencies (Iolascon et al., 2009). Whilst the zebrafish mutant *zinfandel* presents with hypochromic microcytic anemia due to defects in embryonic globin production (Brownlie et al., 2003), hypochromic anemia in the form of congenital sideroblastic anemia in the mutant *sauternes* is caused by disrupted heme biosynthesis (Brownlie et al., 1998). Another disease hallmarked by hypochromic anemia is hemochromatosis. In this disease, erythrocytes are fully functional; however, iron levels in circulation are too low to provide sufficient hemoglobinization. Characterization and positional cloning of the zebrafish mutant *weissherbst* enabled the discovery of a conserved vertebrate iron exporter, Ferroportin 1, whose mutation causes the hypochromic phenotype in this strain (Donovan et al., 2000; Fraenkel et al., 2005). A mutant that shows a very similar phenotype to the one observed in *weissherbst* is the *chianti* strain. Unlike *weissherbst*, the underlying cause is not a lack in circulatory iron, but rather defective iron acquisition due to mutations in the gene encoding Transferrin receptor 1 in differentiating erythrocytes (Wingert et al., 2004).

Finally, the *chardonnay* zebrafish mutant adds another important player to the understanding of iron metabolism, by revealing an essential role of the iron transporter DMT1 in iron homeostasis (Donovan et al., 2002). Moreover, because of its transparency during embryonic development, zebrafish is a very suitable and direct model for porphyrias, which are disorders caused by disrupted heme biosynthesis often accompanied by light sensitivity. The zebrafish *dracula* mutant, which was, like most anemic zebrafish strains, identified in a genetic screen, represents a very accurate model for erythropoietic protoporphyria. The *dracula* gene was shown to encode for Ferrochelatase, the terminal enzyme in the heme biosynthesis pathway, and its inactivation rendered erythrocytes highly light sensitive (Childs et al., 2000). Interestingly, Lenard et al. successfully modeled drug-induced hemolytic and chemotherapy-induced anemia (see poster: Blood toxicity), and used live imaging technologies to visualize *in vivo* hemolysis and regeneration (Lenard et al., 2015).

## Discussion

The blood system is highly conserved between zebrafish and mammals. This high degree of conservation indicates that knowledge obtained from zebrafish is potentially transferrable to humans, and zebrafish models can be used for modeling human blood disorders. The high fecundity and *ex utero* embryogenesis, facilitating non-invasive *in vivo* analyses of zebrafish, enable the application of a wide variety of genetic and drug screening approaches (Box 4), and can make important contributions to our understanding of disease pathophysiology, genotype-phenotype correlations, and eventually enable the discovery of new therapeutic targets and modalities. Limitations that still need to be overcome involve the concurrent and selective expression of oncogenes in adult zebrafish tissues, enabling improved phenocopying of human disorders. In this regard, an interesting novel approach has been recently demonstrated, allowing injection of DNA constructs in adult fish at a certain time point and at any specific location (Callahan et al., 2018). This system, called ‘transgene electroporation in adult zebrafish’ might become useful for hematopoietic diseases, e.g. through injection of DNA constructs with specific hematopoietic promoters into the KM of adult zebrafish. Another important limitation for a wider adoption of zebrafish models is the availability of analysis tools such as reliable antibodies for labeling cell-surface markers to dissect zebrafish hematopoiesis in depth. Such reagents exist for mammalian systems, and their development for zebrafish would facilitate cross-model discovery and translational advances. At the moment, flow-cytometry-based analyses solely rely on forward-sideward scattering (Traver et al., 2003) or on the use of fluorochromes in transgenic lines. Functional assays, however, such as the zebrafish HSC/KM cells methylcellulose colony assays, which allows *ex vivo* characterization of zebrafish hematopoietic precursors (Stachura et al., 2011; Svoboda et al., 2016), further improved the analysis of zebrafish hematopoiesis. However, our knowledge of the zebrafish hematopoietic niche is still sparse and, although more and more studies investigate the interaction between blood cells, their niche and their relevance for blood disorders (Espín-Palazón et al., 2014; Kapp et al., 2018; Konantz et al., 2016; Mahony et al., 2016, 2018; Tamplin et al., 2015), the community needs continued support of basic research. In sum, zebrafish offer unique advantages complementary to mammalian models and promise to greatly facilitate the discovery of new drugs and novel molecular processes involved in healthy hematopoiesis and blood disorders.

**Box 4. Drug screening in zebrafish**

Drug screening of a whole organism allows concurrent observation of drug toxicity and *in vivo* drug effects, and allows the drug to interact with any biological pathway and all respective niches. Owing to their small size and high fecundity, chemical screening in fish is easily feasible and can be performed in a high-throughput manner with different read-outs, such as morphology, behavior and cell state. Morphology screens are designed based on a chosen morphology change of interest. For example, Shafizadeh and colleagues used o-dianisidine staining to detect changes in hemoglobin synthesis after chemical treatment and identified compounds that reduced hemoglobin abundance and as such led to hemolytic anemia (Shafizadeh et al., 2004). Behavior-based screens have also been performed, e.g. by measuring photomotor responses (Kokel et al., 2010). One important chemical screen using whole-mount *in situ* hybridization as a read-out has identified prostaglandin E2 as a novel compound to regulate HSC homeostasis (North et al., 2007). This compound has made it into clinical trials, highlighting the importance of zebrafish in drug screenings (Cutler et al., 2013).
